# Brunei’s teacher education programs: insights into students’ coping and help-seeking strategies to challenges

**DOI:** 10.1186/s13033-016-0091-5

**Published:** 2016-09-29

**Authors:** Lawrence Mundia, Masitah Shahrill, Jainatul Halida Jaidin, Rosmawijah Jawawi, Mar Aswandi Mahadi

**Affiliations:** Sultan Hassanal Bolkiah Institute of Education, Universiti Brunei Darussalam, Jalan Tungku Link, Gadong, Bandar Seri Begawan, BE1410 Brunei Darussalam

**Keywords:** Brunei, Teacher education, Student teachers, Coping, Help-seeking, Achievement

## Abstract

**Background:**

Brunei started implementing its two main reformed teacher education programs, MTeach and MEd, in 2009. The reasons for these innovations included upgrading the standard of teacher training, increasing teaching effectiveness, and improving the quality of education in the country. The purpose of this study was to determine how student teachers coped with and sought help on the challenging programs.

**Methods:**

Using an online survey design, 76 randomly selected recent graduate teachers responded appropriately to questionnaires administered to them by email. The obtained quantitative research information included demographic, coping, and help-seeking data, all analyzed by SPSS Version 22.

**Results:**

Participants endorsed both the productive and nonproductive coping strategies. In addition, they depended more on peers, teachers and internet sources for help. Four major findings were obtained. First, task-oriented coping was the most important and significant predictor of success on the MTeach and MEd programs. Second, females had a higher likelihood of success compared to males (OR = 22.760, 95 % CI for OR = 12.848–40.320). Third, students who consulted relevant internet resources had higher odds for succeeding compared to those who did not (OR = 2.237, 95 % CI 1.196–4.183). Fourth, less-able students who collaboratively worked with the more-able peers were nearly two times more likely to perform better than those who did not (OR = 1.982, 95 % CI 1.082–3.630).

**Conclusions:**

Coping and help-seeking were positively and significantly related to academic achievement on the two Brunei main teacher education programs. Evidence from the present study suggested that vulnerable and at-risk trainee teachers needed appropriate interventions (educational, counseling and psychotherapy) related to effective use of task-oriented coping and seeking help via cooperative learning, internet sources, and teacher consultations,. Further research with interview probes was recommended to gain additional information on the problem and its solutions.

## Introduction, background and setting

Reformation of the current and ongoing Brunei teacher education programs was formulated in 2008 but implemented from August 2009 as a way of increasing teaching effectiveness and improving the quality of education in the country. Since 2009, Brunei has had two main teacher preparation programs, the Master of Teaching degree (MTeach) and Master of Education degree (MEd). Both programs are located in the Sultan Hassanal Bolkiah Institute of Education (SHBIE) at the Universiti Brunei Darussalam (UBD). The MTeach is an initial or pre-service teacher training program. Trainee teachers on the MTeach program will have a bachelor’s degree with in-depth content in a subject teachable in Brunei government schools (e.g. English, mathematics, geography, history, biology, chemistry, and physics) to meet the admission criteria. On the other hand, the MEd is an in-service teacher education program. Student teachers on the MEd program would have some teaching experience to satisfy the selection criteria. Both programs are practical-oriented and take participants a year and half (three semesters) to complete. The terms teacher training and teacher education mean the same thing in the context of the present study and are used interchangeably. Similarly, the words student teachers and trainee teachers also refer to the same thing in the current study and are used alternatively throughout this article. With increasing demand for teachers in both government and non-government schools in Brunei, the number of teacher candidates is projected to rise in the future. Since 2011, the two main teacher education programs (MTeach and MEd) have been graduating a combined average of 70 new teachers each year. Despite this promising success rate, there have been concerns about the few students who fail each year in both programs. Although the number of failures was small in any particular academic year, a trend or pattern was observed which indicated that the higher the number of trainee teachers recruited, the slightly higher number of failures. Based on these and other observations, there was a growing need to identify both the enablers and barriers to students’ success on these two teacher education programs. Such research information would be useful in informing early educational and psychological interventions in support of vulnerable students at risk of failing. Since teacher education was reformed in 2009, the two programs (MTeach and MEd) have not yet been extensively evaluated. There have been suggestions to evaluate several aspects of the programs including their curriculum, assessment strategies, outputs, and employability of the graduates. The problem of the few failing students provided the rationale and justification to conduct the current study on students’ coping styles and help-seeking behaviors, two factors that may impact academic achievement. Research in these areas may contribute solutions to some of the problems students might be having particularly those that are of mental health/emotional nature.

### Why do research on Brunei trainee teachers’ coping strategies?

Previous research [1] defined coping as the “constantly changing cognitive and behavioral efforts to manage specific external and/or internal demands that are appraised as taxing or exceeding the resources of the person” (p. 141). Coping is always necessary whenever humans and other animals are faced with a challenge, adversity, or difficulties. According to another study [2] coping is a complex and multidimensional construct. Thus research has so far identified more than 100 coping mechanisms [3]. In addition, several psychometric scales have been developed to measure coping strategies in children [4], adolescents [5], and adults [6–8]. Some of the inventories are situational or projective [4] while others are paper-and-pencil self-reports [5, 7]. A number of coping styles are measured by each of these questionnaires. Many of these coping modes adequately explain academic achievement or failure. These include positive coping such as behavioral, emotional, and cognitive engagements as well as negative coping like behavioral and cognitive disengagement, behavioral avoidance, and catastrophizing [2, 9–11]. The extent to which Brunei trainee teachers use suitable, diverse and effective coping strategies is unknown due to lack of research. In view of this, the present study assumed that inappropriate coping might be contributing to student teachers’ failing. This allegation or belief provided the rationale and justification for investigating the trainee teachers’ coping styles in the present study.

### Why conduct research on Brunei student teachers’ help-seeking behaviors?

Research shows that people seek help when they are unable to resolve their own problems. However, not all people seek help when problems occur. For example, research indicates that men were less likely than women to seek help from mental health professionals for a variety of reasons [12, 13]. When psychological problems occur, males demonstrate less positive attitudes toward seeking help in order to avoid talking about stressful events and painful feelings because of masculine norms and stigma [13]. Furthermore, men who are engaged in sports or work in the military or police may identify themselves to a higher level with the norms of masculinity and toughness and may not recognize the benefits of seeking professional help such as counseling or psychotherapy [14]. However, not seeking help or being too much dependent on help from others are both harmful personality dispositions. The present study suspected that unsuccessful Brunei trainee teachers did not seek enough appropriate help from relevant sources. This suspicion or notion was the driving force for investigating the student teachers’ help-seeking behaviors in the current study.

### Theoretical framework of the study

The current study was based on the understanding that people (including trainee teachers) who experience stress undergo three processes, namely: primary appraisal or detecting a threat; secondary appraisal or generating a possible solution; and coping or carrying out an intervention [1, 6]. Although stressed individuals (including student teachers) address stress in a variety of ways, the common effective strategies tend to be problem-focused or confronting the stressor head-on and emotion-focused or lowering the resulting emotional distress [7, 8]. We believe that effective interventions are preceded by appropriate assessments to inform mental health professionals. Furthermore, the ability to cope effectively with the distress and to obtain appropriate help were critical to resolving the stressful problem. Furthermore, the

### Objectives of the study

We do not know all the reasons why some students did not perform well on the two main teacher education programs in Brunei. Since no study has yet been conducted to determine the students’ coping and help-seeking behaviors while on the MTeach and MEd programs, the contribution of these two factors could not be ruled out. In view of this, the main purpose of the present study was to identify the coping strategies and help-sources that were related to academic achievement on these two teacher education programs.

## Methods

We briefly describe below the design, participants, instruments, procedures, and data analysis strategies used in the present study.

### Design

This study was an online survey. The current online survey differed from the telephone, postal, or field survey techniques in that the research instruments were directly emailed to the participants using the participants’ university/student email addresses and there was no face-to-face contact between the participants and the researchers.

### Participants

The number of graduate teachers during the academic year when this study was undertaken was about 105. Our original goal was to involve and use all these graduates in the present study. In view of this, emails with attached instruments were send to all the 105 graduates. Unfortunately, only 95 (90 %) of these student teacher graduates still had active and accessible student email addresses at the time of the study and were administered the research instruments online. The emails failed to reach a total of 10 graduates and were returned to senders, the researchers. From the 95 graduates who received the emails with attachments of instruments, we received only 87 completed questionnaires. The final sample was further reduced to 76 by two main factors: (1) receipt of 15 unusable scales that had too many missing values or unanswered items; and (2) removal of four participants who were on the Graduate Diploma in Education (GradDipED) due to the small number (n = 4). Because of the long time it took to get email responses from the respondents, we did not do a double sampling of the non-respondents to determine whether their views were similar or different from those of cooperators (respondents) who returned the completed questionnaires. Despite this constraint, we noted from the literature that when the population of interest was 105, a sample of 76 (72 % or two-thirds of the original population) was considered to be reasonably adequate for research purposes according to sampling tables provided by statisticians [15]. Based on this assumption, we proceeded to analyze the data for the current study. Table 1 shows some of the characteristics of the 76 respondents by gender, age at the time they were still in training, program and graduation year. In addition, the size of the overall sample (N) and subsamples (n) reported in different tables vary due either to pair-wise or list-wise deletion of cases with missing values (depending on the statistical analysis performed by SPSS Version 22 program). Furthermore, the GradDipEd group was excluded from most analyses due to a small number (n = 4).

**Table 1:** Participants’ gender, age, and program of study (N = 76)

Gender	n	Mean age	Std. deviation
Males	16	30.812	6.057
Females	64	31.015	5.298

Program	n	Percentage	Cumulative %
MTeach	38	47.500	52.500
MEd	38	47.500	100.000

### Instruments

Data for the study were collected using three instruments. The first instrument was a researcher-constructed demographic questionnaire that collected self-reported information pertaining to the participants’ gender, age, and program of study. Also collected under the demographic questionnaire were the participants’ self-reported grade point average (GPA) scores to represent academic achievement. Previous research [3, 16] also used GPA scores as a measure of students’ academic achievement. The second instrument was a checklist that collected the participants’ preferred sources of help the student used or consulted most (when faced with difficult academic and personal problems during training). We listed eight (8) sources of help from which participants endorsed all that applied to them. The sources included: the University Counseling Centre, UCC; the Students’ Representative Council, SRC; religion/prayers; the self or self-efficacy; library; computer lab/internet sources; lecturers; and peers. The third instrument used was the adult version of the Coping Inventory for Stressful Situations, CISS **[**7**]** that collected data on the participants’ coping strategies. The CISS scale was a self-report paper-and-pencil measure of coping containing 48 items assessing task-oriented coping (16 items), emotion-oriented coping (16 items), and avoidance-oriented coping (16 items). In the present study, the avoidance scale was further divided into two subscales: distraction (10 items); and social diversion (6 items). Sample items from each of these five subscales cannot be shown here as the CISS is a restricted psychological test whose items’ security, scale validity, and instrument’s commercial value must be legally protected by users. The five scales were all Likert-type instruments each item with a 5-point response format (ranging from 1 = not at all, to 5 = very much). The subscales’ descriptive statistics and reliability indices are presented in Table 2. All the five CISS subscales had satisfactory levels of alpha reliability. Internal consistency reliability estimated by alpha [17] is considered acceptable when in the 0.700–0.800 range [18, 19].

**Table 2:** CISS subscale descriptive statistics and reliability (N = 76)

Subscale	Items	Mean	SEm	SD	Average corrected item-to-scale correlation	Alpha reliability
Task	16	66.111	0.778	7.133	0.524	0.878
Emotion	16	44.366	1.248	11.400	0.635	0.883
Avoidance	16	51.100	1.062	9.604	0.431	0.862
Distraction	10	33.745	0.690	7.165	0.478	0.800
Social diversion	6	19.930	0.444	3.325	0.400	0.634

*SEm* Standard error of the mean

The relationship between the items in each of the five CISS subscales and the whole subscale was assessed by the average corrected item-total correlations (also known variously as non-spurious, attenuated, or adjusted item-to-scale correlations) which were all above 0.400 (see Table 2). The high average item-to-scale correlations provided evidence about the unidimensionality of items in each subscale. In addition, psychometric theory holds that an item measures the same domain as all other items in the same subscale if it correlates positively and highly with total subscale scores of which it does not form a part [20]. The minimum acceptable average non-spurious item-to-scale correlation as evidence of subscale unidimensionality is 0.300 [21, 22].

We also examined the criterion-related validity of the CISS subscales by inter-correlating the subscales within the CISS instrument. Criterion-related validity may also be obtained by correlating subscales in two different instruments that measure the same concept or domain such as the CISS subscales [7] versus subscales in the full version of the COPE Scale [6]. According to the developers of the COPE Scales, the letters “COPE” in the name of this instrument are not abbreviations of other words and do not refer to other words [6]. The inter-subscale correlations in Table 3 formed two groups and may be interpreted in two different ways. Group 1 consisted of low correlations obtained between task, emotion and avoidance subscales. The low correlations in this group suggested that these scales were measures of conceptually and empirically different constructs and did not replicate or repeat each other. For these scales, the low correlations confirm that the questionnaires were distinct from each other and reflect relatively high levels of independence in functioning thereby providing good quantitative evidence for the scales’ discriminant or divergent validity. On other hand, Group 2 comprised of correlations obtained between avoidance, distraction and social diversion subscales. The moderate-to-high significant positive correlations in this group implied that the scales (to some extent) overlapped and measured the same construct. Previous research also found these three subscales to be highly correlated [23]. Indeed all the items for both the distraction and social diversion scales were drawn from the avoidance scale. The paired scales in Group 2 could therefore be said to have satisfactory convergent/concurrent validity. With adequate criterion-related validity, no factor analyses were performed to determine the construct validity of the CISS with the Brunei student teacher sample in the current study. The correlations in Table 3 were sufficient to demonstrate that the CISS was not a unidimensional scale but rather an inventory with three distinct domains (task, emotion and avoidance). Some previous studies that used the CISS elsewhere [24, 25] did not also do any factor analysis when the instrument concerned was an established scale and the investigation was not a validation study.

**Table 3:** Discriminant and convergent validity of the CISS subscales (N = 76)

Scale	Task	Emotion	Avoidance	Distraction
Task	1			
Emotion	−0.020	1		
Avoidance	0.283*	0.172	1	
Distraction	0.188	0.181	0.942**	1
Social Diversion	0.367**	0.094	0.761**	0.903**

* p < 0.05 (two-tailed)

** p < 0.01 (two-tailed)

### Procedures

Regarding the present study’s research ethics, three major points may be noted. First, the participants in the current study were postgraduate adult students who had already graduated and left the university, UBD. In technical and legal terms, the participants were no longer UBD students and therefore not under the jurisdiction of UBD. Based on this argument and the fact that the study was not funded by UBD, the University Ethics Committee at UBD was not obliged to authorise this study although the researchers were UBD academic staff in SHBIE. Second, apart from the MEd participants who were inservice students, the MTeach participants were not yet in employment at the time of conducting the current study. Permission to involve all the 76 participants in the present study could therefore not be obtained from a single source such as the Ministry of Education. Third, the present study was conducted as a small-scale, in-house, and none-funded evaluation of the two teacher education programs (MTeach and MEd) to determine the coping styles and help-seeking strategies students were using. For such small exploratory investigations (including graduate students’ research exercise projects), SHBIE administration (via the Dean’s Office) was permitted by UBD to grant faculty-level authorization to do the studies. In view of this, approval to carry out the current study was obtained from the Dean of SHBIE who was also a co-researcher and co-author.

We emailed the selected participants all the three research instruments and an informed consent form with a brief explanation of the purpose and objectives of the study. On the consent form we also explained the major ethical conditions or requirements for being involved in the study. Only individuals who voluntarily agreed to participate by signing the consent form were recruited into the study. All the instruments (demographical questionnaire, help-sources checklist, and CISS questionnaire) were written in simple or easy English for our sample of graduate students and did not require translation to Bahasa Melayu, the main and official language of Brunei. With regard to the participants’ self-reported GPA scores, these were collected solely for purposes of the present study. In addition, the GPAs were kept confidential and only the researchers had access to them. Furthermore, the GPA scores were only analyzed and reported at the group level to protect the participants’ anonymity.

### Data analysis

Altogether, the data on gender, age, coping and help-seeking were used as independent variables (IVs) when performing various statistical analyses while GPA scores were used as the dependent variable (DV). The five subscales (task, emotion, avoidance, distraction and social diversion) were scored according to instructions in the CISS technical manual **[**7**].** Only raw data were used in the present study. Raw data from the scales were analyzed by a variety of quantitative procedures: descriptive statistics (frequencies, percentages, mean, and standard deviation); and inferential statistics, Pearson correlations, hierarchical multiple regression analysis with backward elimination, and the factorial generalized linear regression model (GLM).

## Results

To address our research aim, we present here the results of the relationship between coping and help-seeking with academic achievement. We used two types of regression analyses, namely: (1) a hierarchical multiple regression analysis with backward elimination; and (2) the factorial generalized linear multiple regression model. The dependent variable (DV) for both of these models was GPA scores. We briefly explain below separately the reasons for performing these analyses and the results from each undertaking.

### The hierarchical multiple regression analysis with backward elimination

The purpose of performing a hierarchical multiple regression analysis with backward elimination was to explore and identify continuous, dichotomous and multi-categorical independent variables (IVs) that were related to the dependent variable (DV). For security and ethical reasons, we chose to use the no constant model (linear regression through the origin) to prevent a possible reconstruction of GPA scores through a multiple regression equation. In multiple regression, the unstandardized or standardized regression coefficients give the estimated change in the response variable (DV) associated with a unit change in the corresponding explanatory variable (IV), while holding all the other independent variables constant. The less stringent default significance level required by SPSS [26] and SAS [27] for variables to enter and stay in the regression model/equation is p = 0.15. However, the present study used p = 0.05 for entry and p = 0.10 for removal.

### Results from hierarchical multiple regression analysis with backward elimination

Table 4 presents the first required output from the analysis showing the change statistics that occurred to F, R, R^2^, Adjusted R^2^, SE, ΔR^2^, ΔF, and significance level for ΔF from model 1 to model 5. As usual, each step was a model and each of the five models was significant because of the inclusion of one or more variables at each stage that were significantly related to the DV, achievement. However, only the ΔF for model 1 was significant because no significant changes occurred to R, R^2^, Adjusted R^2^, ΔR^2^, and ΔF in models 2–5.

**Table 4:** Variable selection using hierarchical multiple regression analysis with backward elimination (N = 76)

Model	df	F	R	R^2^	Adj R^2^	SE	ΔR^2^	ΔF	df1	df2	Sig. ΔF
1	756	391.524**	0.990	0.980	0.977	0.551	0.980	391.524	7	56	0.000**
2	657	464.858**	0.990	0.980	0.978	0.546	0.000	0.009	1	56	0.924
3	558	567.087**	0.990	0.980	0.978	0.541	0.000	0.052	1	57	0.820
4	459	719.182**	0.990	0.980	0.979	0.538	0.000	0.150	1	58	0.700
5	360	957.200**	0.990	0.980	0.979	0.538	0.000	1.084	1	59	0.302

** p < 0.01 (two-tailed)

The variables that were entered originally at step 1 and those that were retained in subsequent stages in the 5-step backward elimination model are presented in Table 5 together with the regression coefficients (unstandardised and standardised), t values and significance levels. The variable “Avoidance” was excluded from analysis as it correlated too high with both “Distraction” and “Social diversion” variables (see Table 3 above). The SPSS analysis rejected “Avoidance” as a redundant variable. In view of this, “avoidance” was excluded in Table 5. The other dichotomous IV that was, for ethical reasons, excluded from Table 5 analyses was “program”. When the article was read by the participants and subsequent cohorts of teacher trainees, we did not want to create or give an impression through such direct comparisons that one group (e.g. MTeach or MEd) was better or superior in coping and seeking help while the other was inferior. The findings in Table 5 indicate that gender (a dichotomous variable) and task-oriented coping (a covariate) were the only predictors of success (GPA) on the MEd and MTeach programs with our study sample. However, we could not tell from this table which of the two genders was most significantly related to academic achievement. We also noted from Table 5 that the multi-categorical variable “helpers” (sources of help or help-seeking) approached the significance level. This implied that one or more of the eight different help sources embedded in this variable might be significantly related to academic achievement.

**Table 5:** Hierarchical multiple regression analysis with backward elimination on GPA scores (N = 76)

Model	Variables^‡^	Unstandardized coefficients		Standardized coefficients	t	Sig.
		B	Std. error	Beta		
1	Gender	0.425	0.165	0.213	2.579	0.013*
	Age	0.014	0.013	0.117	1.050	0.298
	Task	0.026	0.010	0.460	2.675	0.010*
	Emotion	0.001	0.006	0.015	0.193	0.848
	Distraction	0.001	0.012	0.010	0.095	0.924
	Social diversion	0.006	0.024	0.033	0.249	0.804
	Helpers^a^	0.081	0.055	0.152	1.476	0.146
2	Gender	0.427	0.161	0.214	2.649	0.010*
	Age	0.014	0.013	0.117	1.064	0.292
	Task	0.026	0.009	0.461	2.723	0.009**
	Emotion	0.001	0.006	0.017	0.228	0.820
	Social diversion	0.007	0.022	0.038	0.325	0.746
	Helpers^a^	0.080	0.054	0.151	1.486	0.143
3	Gender	0.432	0.158	0.217	2.729	0.008**
	Age	0.014	0.013	0.118	1.073	0.288
	Task	0.026	0.009	0.467	2.806	0.007**
	Social diversion	0.008	0.021	0.044	0.387	0.700
	Helpers^a^	0.082	0.053	0.154	1.543	0.128
4	Gender	0.443	0.155	0.222	2.861	0.006**
	Age	0.013	0.013	0.112	1.041	0.302
	Task	0.028	0.008	0.500	3.534	0.001
	Helpers^a^	0.088	0.051	0.164	1.715	0.092
5	Gender	0.452	0.155	0.226	2.920	0.005**
	Task	0.033	0.006	0.594	5.469	0.000**
	Helpers^a*†*^	0.094	0.051	0.177	1.856	0.068

^a^ *Helpers* sources of help used when participants sought assistance (this variable had eight categories)

* p < 0.05 (two-tailed)

** p < 0.01 (two-tailed)

^†^Neared the significance level at p = 0.05

^‡^Avoidance was brocken into two variables—distraction and social diversion—was omitted from analysis as it correlated too high with both distraction and social diversion (see Table 3 above)

### The factorial generalized linear multiple regression model

To identify the specific gender and help sources that were most related to academic achievement, we performed a factorial generalized linear regression model analysis (GLM) using gender and help-sources as IVs and GPA scores as the DV. There were four main reasons why we analysed the data using the factorial GLM. First, the factorial GLM is mathematically identical to a multiple regression analysis but is suitable for both qualitative and quantitative IVs that are coded dichotomously and multicategorically. Our IVs, gender and help-sources had two and eight categories respectively while the DV, GPA, was continuously scored. Second, the factorial GLM aims to determine (explain or predict) the variation in a dependent variable in terms of a linear combination or weighted sum of several reference functions. Third, for small samples, the t values in multiple regression analysis are not valid and Wald’s (X^2^) statistic should preferably be used instead. Wald’s statistic (defined as the ratio of the square of the regression coefficient to the square of the standard error) is analogous to the *t* test in linear multiple regression and is basically a t^2^ that is asymptotically distributed as Chi square with df = 1. Given that N = 76 in our analysis (MTeach and MEd groups combined), our sample was not large but moderate (though not too sparse for the findings to be biased). Fourth, a factorial GLM analysis provides exponentiated regression coefficients that are interpreted as odds ratios. In this way, the factorial GLM was a good alternative to the binary and multinomial logistic regression analyses that require dichotomous and multicategorical DVs respectively. The results of our factorial GLM analysis are presented in Table 6. It can be noted from this table that both the regression coefficients and their corresponding standard errors were low thereby reducing the probability of a Type-II error from occurring.

**Table 6:** Impact of gender and help-sources on academic achievement using a factorial generalized linear regression model on GPA scores (N = 76)

Factors	B	Std. error	95 % CI for B		Hypothesis test			OR	95 % CI for OR	
			Lower	Upper	Wald X^2^	df	Sig.		Lower	Upper
UCC	–	–	–	–	–	–	–	–	–	–
Males	2.875	0.322	2.244	3.506	79.730	1	0.000***	17.722	9.429	33.310
Females	3.125	0.291	2.553	3.697	114.719	1	0.000***	22.760	12.848	40.320
SRC	–	–	–	–	–	–	–	–	–	–
Religion/prayers	0.195	0.523	−0.831	1.221	0.139	1	0.709	1.216	0.436	3.390
Self (self-efficacy)	0.408	0.359	−0.296	1.111	1.289	1	0.256	1.503	0.744	3.038
Library	0.311	0.345	−0.366	0.988	0.812	1	0.368	1.365	0.694	2.685
Computer lab/internet	0.805	0.319	0.179	1.431	6.355	1	0.012**	2.237	1.196	4.183
Lecturers^†^	0.510	0.306	−0.090	1.109	2.776	1	0.096	1.665	0.914	3.033
Peers	0.684	0.308	0.079	1.289	4.906	1	0.027*	1.982	1.082	3.630

*UCC* University Counseling Centre (not endorsed by any participant), *SRC* Student Representative Council (not endorsed by any participant)

* p < 0.05 (two-tailed)

** p < 0.01 (two-tailed)

*** p < 0.001 (two-tailed)

^†^Neared the significance level at p = 0.05

### Results from the factorial generalized linear multiple regression model

Three major findings emerged. Gender was significantly related to academic achievement. However, compared to males, the females had a higher likelihood of success and were 23 times more likely to perform better than males (OR = 22.760, 95 % CI for OR = 12.848–40.320). Second, computer lab/internet (as a source of help) was also significantly related to academic achievement. Students who consulted relevant internet resources had higher odds for succeeding and were 2 times more likely to perform well compared to those who did not (OR = 2.237, 95 % CI 1.196–4.183). Third, peers were an important help resource in facilitating academic achievement (p < 0.05). Students who collaboratively worked with more-able peers were 2 times more likely to perform well than those who did not (OR = 1.982, 95 % CI 1.082–3.630). Fourth, lecturers were also important resource persons in promoting academic achievement (p < 0.05). Students who consulted lecturers were nearly 2 times more likely to perform well than those who did not (OR = 1.665, 95 % CI 0.914–3.033). All the odds ratios for the above four variables (gender, computer lab/internet, peers, and lecturers) had 95 % CI lower limits that were either close to 1 or greater than 1 in magnitude, an indication that they were statistically significant at p = 05 (Table 6). Overall, we obtained altogether four IVs that were strongly related to academic achievement and predicted success on the MTeach and MEd programs, namely: gender; task-oriented coping; computer lab/internet sources; and peers (see Tables 5, 6).

## Discussion, implications and recommendations

The survey investigated the coping strategies and help-seeking behaviors of Brunei student teachers. Based on the major findings of the study, we briefly discuss below the main implications derived and make recommendations to address concerns arising from them.

### Productive and nonproductive coping strategies

Of all the five CISS coping strategies, task-oriented coping was the only one that was associated with academic achievement. In the context of the CISS questionnaire [7], task-oriented coping was an example of productive coping. This was the best, most effective, and highly desired form of coping designed to confront the distress head-on. Task-oriented coping was the equivalent of productive coping in the adolescent coping scale [5] and active coping in the COPE scale [6]. In the literature, it is also generally referred to as behavioral engagement coping [2, 10, 11] or as proactive coping [28]. In the current study, students who used the task-oriented coping style had higher likelihood of succeeding. Literature indicated that productive coping was correlated with academic achievement or GPA and with personality traits such as conscientiousness, openness and agreeableness [3]. If used properly and efficiently, task-oriented coping had potential to help all categories of students to achieve higher academic results. Since recent relevant literature suggests that coping strategies are alterable and teachable [2, 10, 11, 29], we recommend that motivational talks, seminars and workshops be conducted to provide group training to distressed MTeach and MEd students on the effective use of task-oriented coping strategies, peer learning, internet sources, and lecturer consultations. All the other forms of CISS coping strategies (e.g. emotion-oriented, avoidance-oriented, distraction-oriented, and social diversion-oriented) on which our participants scored high, were not highly and significantly correlated with academic achievement, GPA. These are the examples of none-useful coping strategies in the present study. Other researchers similarly found some Asian students scoring high on these dysfunctional coping strategies [30–32]. The highest scoring students on these none-effective coping dimensions need to be identified and persuaded to undergo appropriate voluntary counseling (in an individual or group setting) to learn more about using task-oriented coping strategies, peer learning, internet sources, and lecturer consultations. These are the students with high support needs. The present study found avoidance, distraction, and social diversion coping variables as inhibitors or barriers to high achievement.

### The self or self-efficacy

The eight sources of help that we posed to participants to choose from and endorse included the self or self-efficacy (SE). The findings from this study suggested that most of the participants in the sample were more dependent on external sources for solutions to their problems. Although seeking help from people (e.g. peers and lecturers) is encouraged and evidence from the current study showed that struggling students benefitted from this, the use of internal resources such as the self or self-efficacy must also be concurrently fostered and encouraged in trainee teachers and other university students to eventually promote long-term self-help, independence, and autonomous attitudes in solving learning and personal problems (rather than resorting to depending on others always for help). SE is especially an essential, desirable and important psychological factor or trait that needs to be developed in student teachers because it empowers them to own their problems and devise self-care strategies. The development of SE in students might contribute to the growth of other self-related attributes such as self-regulation, self-direction, self-motivation, self-esteem, and self-management, all of which promote a sense of feeling self-worth in an individual. In addition, previous research has found SE to be a significant correlate and predictor of academic achievement [33, 34]. Furthermore, previous research has also found high SE scores to be positively and significantly related with productive coping scores while low SE scores correlated with procrastination, buck-passing, and nonproductive coping strategies [34]. Moreover and above all, SE is correlated with psychological dispositions that help students to perform better such as behavioral engagement, emotional engagement, and cognitive engagement [10, 11]. Efforts in future research should therefore be directed at investigating the various ways SE could be built up in Brunei student teachers and other tertiary students.

### The University Counseling Centre

An important source of help that was most underused by the participants in the present study was the University Counseling Centre (UCC). None of the participants endorsed UCC as a help outlet and in the absence of interviews, it was not clear from the current study why this facility was neglected by the participants. Since the findings from this study indicated that students benefitted when they received help from other people (e.g. peers and lecturers), future mixed-methods research (that incorporates interviews with probes) should be carried out to shed light and insights on how students might be encouraged to seek counseling. The participants either did not know the benefits of undergoing counseling (both referred and voluntary) or they under-rated the type and quality of services offered by the UCC. Students may also have unfounded fears of the stigma associated with visiting a mental health clinic for professional help. In addition, they may be shy, cautious and apprehensive about seeing mental health professionals. Part of the solution to this dilemmatic problem of disinterest in seeking professional help may require conducting a message awareness campaign to sensitize students about the value of using services offered by pastoral care outlets such as the UCC. It may also be in the best interests of the students if the university considered the possibility and viability of training and using student peer counselors. This is because students seem to trust peers more than counselors and psychologists and probably feel freer to approach or confide with fellow learners.

### Most widely used sources of help

The main sources of help that were endorsed most by students in the current study were computer lab/internet resources and peers. Both of these variables were significantly related to academic achievement in the present study. Students who used computer lab/internet sources had the highest likelihood to perform well (OR = 2.237, 95 % CI 1.196–4.183) followed by those who consulted peers or worked collaboratively with other students (OR = 1.982, 95 % CI 1.082–3.630). The variable, lecturers, approached the significance level thereby showing a trend for it being useful and important in the current study (OR = 1.66, 95 % CI 0.914–3.033). These findings implied that the student teachers with high support needs should use more internet sources, peers and lecturer resources for help (and not less) as they simultaneously try to develop their own self-efficacy. Students in other previous Asian studies also cited external attributions such as peers and lecturers as sources of help for success [32].

## Conclusion

This study investigated the coping strategies and help-seeking behaviors of trainee teachers. Participants scored high on all the CISS coping strategies suggesting that they utilized all the five coping mechanisms. Task-oriented coping, the best approach for withstanding distress, was the only significant predictor of academic success. Findings revealed the participants’ heavy dependence on the use of peers and internet for help. Both variables were predictors of academic achievement. Less emphasis was placed on utilization of the self or self-efficacy. For unknown reasons, the University Counseling Centre and the Students’ Representative Council were not endorsed by the participants as a major external source of help. Further mixed-methods research which includes interview probes was recommended to provide insights on: (1) how participants utilize coping strategies and sources of help; and (2) types of educational, counseling, and psychotherapy interventions that would be most beneficial to vulnerable students at risk of failing due to employing dysfunctional coping and help-seeking strategies.

### Limitations of the study

The present study had two main limitations. First, it had no interview qualitative component to probe some of the responses from the self-report quantitative survey. Second, the final sample size for the online survey was reduced to 76 participants largely by non-response bias. This was mainly due to the fact that some of the selected graduates had defunct email addresses and either did not receive the emailed survey instrument or declined to participate. Despite these constraints, the study may be of interest and value to teacher educators and educational researchers.

## Abbreviations

UBD: Universiti Brunei Darussalam
SHBIE: Sultan Hassanal Bolkiah Institute of Education
MTeach: Master of Teaching
MEd: Master of Education
CISS: coping inventory for stressful situations
GradDipEd: Graduate Diploma in Education
GPA: grade point average
GLM: generlised linear regression model
SPSS: statistical package for the social sciences
OR: odds ratios
SE: self-efficacy
UCC: University Counseling Centre
SRC: Student Representative Council
